# Supplementary material for: Scalable sequence database search using partitioned aggregated Bloom comb trees
Source: Bioinformatics. 2023 Jun 30;39(Suppl 1):i252–9. doi: 10.1093/bioinformatics/btad225 (PMC10311332; doi:10.1093/bioinformatics/btad225)
Supplement: btad225_Supplementary_Data [file btad225_supplementary_data.pdf]

1 Appendix

1.1 Definitions

A *Bloom filter* (BF) is a space-efficient data structure for inexact set representation. For Bloom filters, inexact means that queries to such a representation have a controlled rate of false positives and no false negatives. A Bloom filter has two parameters (b, h), which define the size of a bitvector and the number of hash functions of the filter. A Bloom filter maps each set element in the bitvector using h hash functions, and sets mapped bits to 1 (initially, all bits are 0's). The query follows the same principle and verifies that all hashed locations contain a 1. False positives on foreign keys occur because of hash collisions (which can be controlled by tuning (b, h)).

A *minimizer [1]* of size m of a $k-$mer S $(m < k)$ is the smallest hashed substring of size m in S.

Proof of observation 1

We define a series of Bloom filters BF_1, BF_2, $\ldots BF_t$ built using common (b, h) parameters. They represents k-mer sets $R_1, R_2, \ldots, R_t$ such that $R_t \subseteq \ldots R_2 \subseteq R_1$. We encode this particular series by viewing it as a matrix M of size $t \times b$. We recall that a SBT is built using the merge operation on pairs of leaves to obtain a binary tree.

Proof 1 *One can notice that by recursion, any bit set to 1 in* $BF_i, (1 < i \leq 2)$ *is also set to 1 in* $BF_{i-1}, \ldots BF_1$. *Similarly, if a bit is set to 1 in a Bloom filter of a SBT, the 1 is conserved through the recursive OR operations that build the parent nodes of this Bloom filter.*

1.1.1 Implementation details

K-mer representation. Each k-mer is represented by the lexicographically smallest of the forward string and reverse-complemented string (canonical k-mer) and is hashed using a fast xorshift hashing function.

Figure S1 presents the partition scheme based on minimizers and super-k-mers.

References

[1] Michael Roberts, Wayne Hayes, Brian R Hunt, Stephen M Mount, and James A Yorke. Reducing storage requirements for biological sequence comparison. *Bioinformatics*, 20(18):3363–3369, 2004.

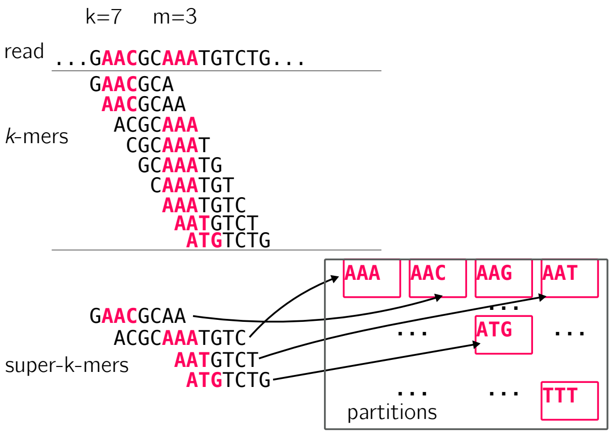

Figure S1: Example of minimizers and super-k-mers computed from a read ($k = 7, m = 3$). Minimizers are represented in pink (for the sake of simplicity, we consider the lexicographic order on minimizers). See how the second super-k-mer aggregates several k-mers having AAA as a minimizer. We show partitions corresponding to minimizers in the same color.
